# Cumulative lifetime stressor exposure impairs stimulus–response but not contextual learning

**DOI:** 10.1038/s41598-024-62595-x

**Published:** 2024-06-07

**Authors:** Mario Rosero-Pahi, Jamila Andoh, Grant S. Shields, Alida Acosta-Ortiz, Sergio Serrano-Gomez, George M. Slavich

**Affiliations:** 1 Cognitive and Translational Neuroscience Lab, Faculty of Health Sciences, Universidad Autónoma de Bucaramanga, Bucaramanga, Santander Colombia; 2 Department of Cognitive and Clinical Neuroscience, Central Institute of Mental Health, Medical Faculty Mannheim, Heidelberg University, Mannheim, Germany; 3 Department of Psychiatry and Psychotherapy, Medical Faculty Mannheim, Central Institute of Mental Health, University of Heidelberg, Mannheim, Germany; 4 Department of Psychological Science, University of Arkansas, Fayetteville, AR USA; 5 Department of Psychiatry and Biobehavioral Sciences, University of California, Los Angeles, CA USA

**Keywords:** Cognitive neuroscience, Learning and memory, Quality of life

## Abstract

Greater exposure to stressors over the life course is believed to promote striatum-dependent over hippocampus-dependent learning and memory processes under stressful conditions. However, little research in this context has actually assessed lifetime stressor exposure and, moreover, it remains unknown whether greater cumulative lifetime stressor exposure exerts comparable effects on striatum-dependent learning and hippocampus-dependent learning in non-stressful contexts. To investigate this issue, we used the Stress and Adversity Inventory for Adults (Adult STRAIN) and Multicued Search Task to investigate the relation between cumulative lifetime stressor exposure and striatum-dependent stimulus–response learning and hippocampus-dependent contextual learning under non-stressful conditions among healthcare professionals (N = 205; 157 females, 48 males; Age: M = 34.23, SD 9.3, range 20–59 years). Individuals with moderate, but not low, cumulative lifetime stressor exposure exhibited impaired learning for stimulus–response associations. In contrast, learning for context associations was unrelated to participants' lifetime stressor exposure profiles. These results thus provide first evidence that cumulative lifetime stressor exposure may have negative consequences on human striatum-dependent stimulus–response learning under non-stressful environmental conditions.

## Introduction

Stress refers to a state of threatened homeodynamic balance by a wide range of intrinsic or extrinsic, real or perceived challenges or stimuli, defined as stressors^1–4^. Prolonged exposure to major life stressors exerts substantial effects on the basal activity and responsiveness of the stress system that can lead not only to neuropsychiatry, neurodegenerative, cardiovascular, endocrine, metabolic, autoimmune, and allergic disorders, but also alterations to several cognitive processes including attention, learning, and memory^2,4,5^. Although one major area of progress in stress research has been in determining the effects of early, chronic (i.e. prolonged exposure to a given stress condition), and acute (i.e., stress that is applied transiently) stressor exposure on the brain, cognition, and health, more recent attention has been placed on identifying the consequences of cumulative lifetime stressor exposure on these outcomes^6–12^. Cumulative lifetime stressor exposure is defined as the sum of all acute life events and chronic difficulties that a person has experienced over his or her entire life. Findings from several studies indicate that chronic stressor exposure throughout one’s lifetime is related to hypothalamic–pituitary–adrenal (HPA) axis hypo-activity, resulting from a compensatory adaptation to sustained periods of HPA axis hyper-reactivity^4,13,14^. This downregulation has been interpreted as an improved negative feedback regulation of the HPA axis and involves important neuroendocrine changes, including lower circulating glucocorticoid levels (cortisol in humans and corticosterone in rodents), altered mineralocorticoid receptor (MR) and glucocorticoid receptor (GR) expression and sensitivity and blunted stress reactivity (i.e., capacity to respond to a stressor) of the HPA axis^4,15–17^). Importantly, some studies have shown that greater cumulative lifetime stressor exposure is related to both blunted cortisol response to stressors^6,10,18^, and blunted cortisol awakening response^19^. Which suggests that cumulative lifetime stressor exposure could alter optimal HPA axis activity, resulting in enhanced negative feedback inhibition of the HPA axis, decreased cortisol response and reduced basal cortisol secretion.

In addition to the effects of cumulative stress exposure on glucocorticoid availability and signaling, converging evidence from rodent and human studies indicate that the engagement of striatum-based and hippocampus-based memory systems may be influenced by lifetime exposure of stress, and that this influence is mediated by glucocorticoid signaling. In dual solution tasks (i.e., tasks that can be solved by different memory systems), induction of acute or chronic stress has been shown to promote a shift from hippocampus-dependent learning to striatum dependent learning^20–24^ via glucocorticoids^25–27^. Importantly, pre-learning stress has been related to impairment of hippocampus-dependent memory^28–30^. On the other hand, under non-stressful environmental conditions, lower cortisol levels preferentially lead to the engagement of hippocampal over striatal learning strategies, while moderate glucocorticoid elevations have the opposite effect^26,27^. Similarly, it has been reported that glucocorticoid administration in non-stressed individuals prior to learning enhanced striatum dependent stimulus–response S–R learning^31^. Furthermore, glucocorticoid administration prior to learning impairs hippocampus-dependent memory processes^32,33^. Thus, the engagement of the striatal and hippocampal learning systems depends on the stress levels, HPA axis stress reactivity, and basal (non-stress) cortisol concentrations. In the functional domain, hippocampal and striatal memory systems can operate independently^34,35^, synergistically^36–38^ and competitively^39,40^.

Regarding cumulative stress exposure effects on memory systems, some studies suggest that low levels of lifetime stress, impair striatum-dependent S–R learning, while hippocampus-dependent learning performance remain relatively unaltered^41^. Conversely, chronic exposure to stress impairs hippocampus-dependent learning^42–44^ and promotes the engagement of striatum-dependent S–R learning^44^. Although cumulative exposure to stress over a long period exerts substantial effects on the basal activity and responsiveness of the stress system, which in turn can negatively impact brain systems that support learning and memory, few studies have examined long-term effects of cumulative lifetime stressor exposure on the engagement of striatum and hippocampus-dependent learning regarding basal (i.e., unstressed) conditions.

Importantly, healthcare professionals are more frequently exposed to occupational stress factors, including high workloads, long shifts, nonstandard work schedules, administrative burdens and incivility between coworkers^45–48^. Notably, several studies have found that occupational stress exposure can result in burnout, anxiety, depression and post-traumatic stress disorder^48–50^. Similarly, it has been reported that occupational stress exposure can negatively affect cognitive performance^51–53^ and memory^52,54^. Consequently, work-related stress increases the risk of committing medical and administrative errors in daily practice situations and failing to identify life-threatening signs and symptoms^55^. Whereas research on stress among healthcare professionals is abundant, no studies have examined the long-term effects of cumulative lifetime stressor exposure on contextual and S–R learning in healthcare professionals.

The aims of the present study were (1) to describe the mental and physical health, demographic and lifetime stressor exposure features of the healthcare professionals' cohort, (2) to identify latent trajectories of lifetime stressors across the lifespan and explore whether latent trajectories of lifetime stressors are associated with stress groups and (3) to investigated how cumulative lifetime stressor exposure relates to striatum-dependent stimulus–response learning and hippocampus-dependent contextual learning during non-stressful conditions in healthcare professionals. We assessed participants’ cumulative exposure to several different types of stressors that they could have experienced over the entire life course using the Stress and Adversity for Adults (Adult STRAIN)^9^. In addition, we measured their S–R and context learning using the Multicued Search Task, in which the incorporation of S–R and context memory cues results in the formation of representations that can be used to guide attention. Importantly, contextual memory-guided attention critically relies on the hippocampus^56–58^, and S–R and contextual memory-guided attention has been linked to greater striatal and hippocampal blood oxygenation level dependent activity, respectively^59^. Based on previous findings showing that cumulative lifetime stressor exposure can lead to reduced cortisol response^6,10,18^ and decreased cortisol availability^19^, and considering the preferential engagement of hippocampal over striatal learning strategies under both non-stressful conditions and lower cortisol levels^26,27^, we expected that individuals with moderate, but not low cumulative lifetime stress, would exhibit impaired learning for S–R associations, while the memory for context associations would not be affected by moderate or low cumulative lifetime stress. However, because previous studies on the effects of chronic stress on memory suggest that chronic exposure to stress impairs hippocampus-dependent learning and facilitates striatum-dependent S–R learning^44^, it was difficult to predict the effects of cumulative lifetime stressor exposure on S–R and contextual learning.

## Method

### Participants

Participants were 205 full-time healthcare professionals (157 females, 48 males; Age: M = 34.23, SD 9.3, range 20–59 years), with no history of neurological or mental disorder, recruited via e-mails, from five hospital units (i.e., Intensive Care, Emergency, Surgery, Hospitalization Unit, and General Outpatient Department) at Fundación Oftalmológica de Santander (FOSCAL), Colombia. The Research Ethics Committee of the Fundación Oftalmológica de Santander approved the study, and written informed consent was obtained from all persons before participation. This study was performed in accordance with the ethical standards of the 1975 Declaration of Helsinki and its later amendments in 2013.

### Stress and adversity inventory for adults (adult STRAIN)

A Spanish version of the Adult STRAIN^9^ was used to assess participants’ cumulative exposure to several types of acute and chronic stressors that they could have experienced over the entire life course (see https://www.strainsetup.com). The Adult STRAIN was forward translated from English to Spanish and then back translated according to established procedures. Specifically, the Adult STRAIN assesses exposure to 55 different lifetime stressors, including 26 acute life events and 29 chronic difficulties. These stressors span two stress exposure indices, two stress exposure timing categories, two stressor types, 12 primary life domains, and five core social-psychological characteristics. The Adult STRAIN assesses the severity, frequency, timing, and duration of each stressor that is endorsed. The Adult STRAIN takes about 18 min to complete, has excellent psychometric properties, and has been well-validated against numerous cognitive, biological, and clinical outcomes^60–63^. Although we described some of those indices in our analysis, the main stress variable used in analyses was participants' total lifetime stressor count, a reliable measure of the objective aspects of the stress that individuals experience^9^. We used the total lifetime stressor count because it includes both acute and chronic stressors, and offers a more complete view of stressors experienced over the life course. The total lifetime stressor count was calculated as the sum of the stressor frequencies. The total lifetime stressor count can range from 0 to 159. In addition, cumulative life stressor severity is calculated as the sum of the perceived severities of all reported stressors, and can range from 0 to 275. Reported stressors can also be classified according to 12 primary life domains (Reproduction, Legal/Crime, Possessions, Work, Education, Financial, Treatment/Health, Housing, Death, Life-Threatening Situations, Other Relationships and Marital/Partner) and five core social-psychological characteristics (Entrapment, Humiliation, Role Change/Reversal, Physical Danger and Interpersonal Loss).

### General mental and physical health complaints

We used the Kessler 6-Item Psychological Distress Inventory (K-6)^64^ and the Physical Health Questionnaire (PHQ)^65^ to assess mental health and physical health, respectively, over the past month. The K-6 is a brief 6-item scale that measures non-specific psychological distress (i.e., as opposed to disorder-specific psychiatric diagnoses). The scores for all items were summed (range = 6–30), with higher scores indicating greater mental health complaints. The PHQ is a 14-item scale that assesses a variety of physical and somatic symptoms, including headaches, upset stomach, and colds. The scores for all items were summed (range = 12–98), with higher scores indicating greater physical health complaints. The PHQ shows good convergence with general health and divergence with work stress^65^.

### Multicued search task

**Figure 1:**
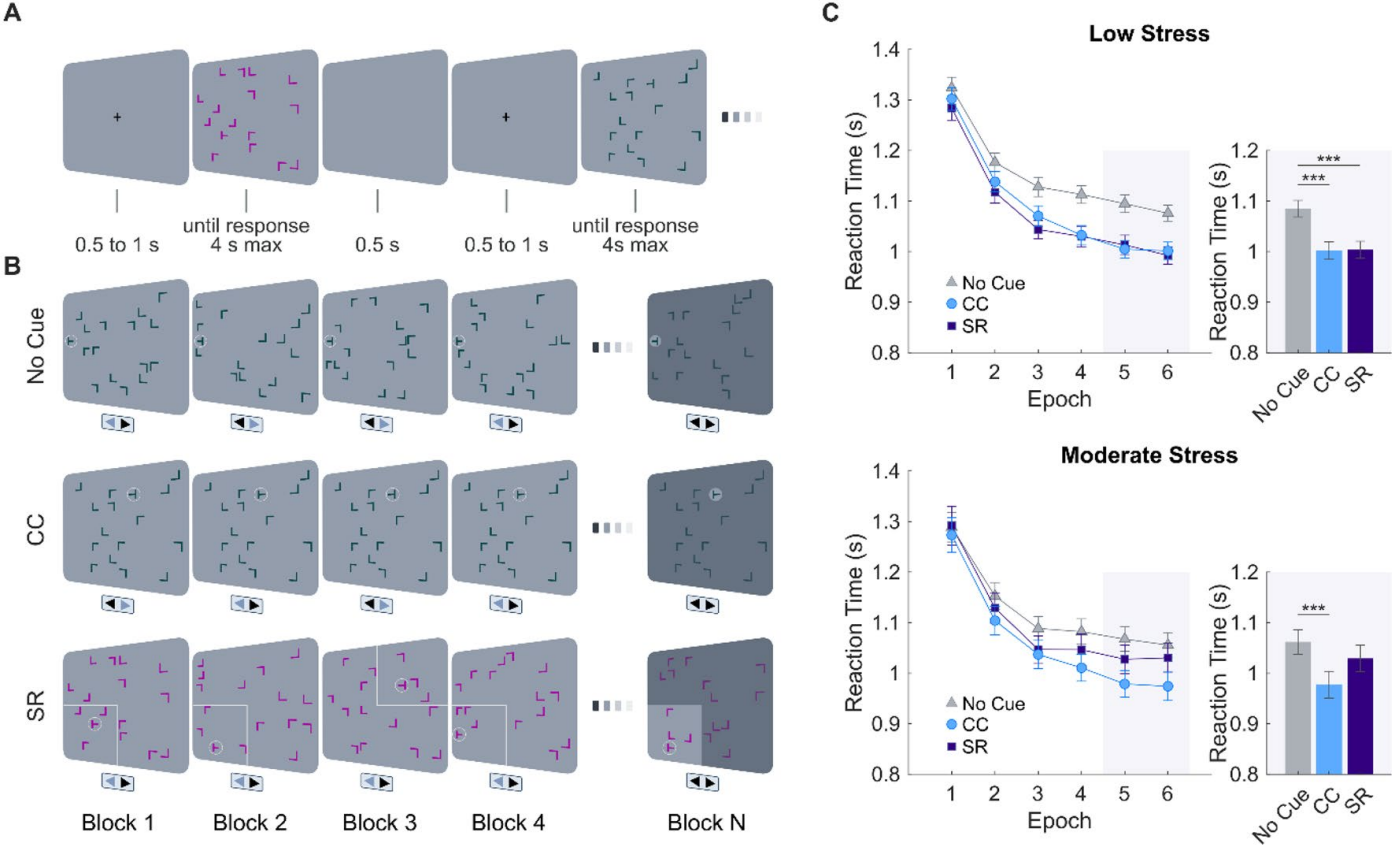
Experimental paradigm. (**A**) Representative sequence of trials with their specific timing parameters. (**B**) Illustration of SR (stimulus–response), CC (context cue), and No Cue trials across the blocks. (**C**) CC and SR learning. Left: mean correct response times RTs (Mean ± 1 SEM) for the low lifetime stressor exposure group (top) or the moderate lifetime stressor exposure group (bottom) as a function of epoch and cue type. Right: mean correct RTs (Mean ± 1 SEM) for the low lifetime stressor exposure group (top) and moderate lifetime stressor exposure group (bottom) as a function of cue type during the last two epochs of the experiment (gray background). Error bars, ± 1 SEM. **p < 0.0, 1***p < 0.001.

Participants completed a version of the Multicued Search Task^59^ implemented and executed in MATLAB (2020a, Natick, MA, USA). In the Multicued Search Task, the participants were encouraged to search for a target item embedded in a spatial array of distractor items. The target was a T stimulus rotated 90 degrees to the right or to the left. The distractor stimuli were L-shapes presented randomly in 1 of 4 orientations (0 degrees, 90 degrees, 180 degrees, 270 degrees). Each display consisted of 16 items (a single target and 15 distractors) randomly positioned in an invisible 12 × 8 matrix (37.2 degrees × 28.3 degrees). For No Cue trials, the distractor configurations were newly generated in each block. On CC trials (contextually cued), the repeated configuration of distractors across blocks cued the target location but did not cue the orientation of the target. In SR trials (probabilistic SR association), the color of the items (blue or pink) across blocks cued (80% probability) the location of the target and the orientation of the target, Fig. 1B. Configurations of target and distractors were randomly generated for each participant. Visual stimuli were presented on a gray background on a 17-inch Dell E2011H LCD monitor. Participants were seated 50 cm in front of the computer monitor. The subjects were told to press 1 of the 2 buttons (“C” or “M”) on a computer keyboard based on whether the bottom of the T was pointed to the right or to the left. Participants were not informed about the different trial types. Each trial consisted of a fixation cross (1–1.5 s), followed by a display presentation (for a maximum of 4 s). This was then followed by a feedback screen (0.5 s), displaying points earned during the previous search (points were calculated based on RT and accuracy, with 1–10 points received on accurate responses, 0 for missed responses, -10 for inaccurate responses). Total points earned were shown at the end of each block.

An example of the trial sequence is shown in Fig. 1A. Each subject performed 24 blocks (576 trials) of the Multicued Search Task with each block containing 24 intermixed trials of 11 No Cue, 8 CC, and 5 SR (4 valid and 1 invalid) displays. Importantly, in the invalid SR display, the cue does not predict the quadrant and pointing direction of the target. There were rest periods of 10 s between blocks.

After block 24, the participants completed two blocks of the explicit recognition test to assess awareness of these mnemonic associations^59,66^. In block 1, participants viewed screens from which the target had been removed and replaced with a distractor. Participants were instructed to indicate the quadrant in which the target appeared when they viewed the screen before responding with 1 of 4 keys. In block 2, participants were instructed to indicate the direction the target was pointing by responding with 1 of 2 keys. Importantly, participants were not informed at the beginning of the experimental session about the recognition test.

### Data analyses

#### Latent class analysis

The underlying distribution of the overall lifetime stressor count was assessed with a latent class analysis using the mclust package, version 5.4^67^. By means of the expectation–maximization algorithm, one through nine Gaussian clusters were fit to the lifetime stressor count data, both assuming equal variance and not, modeled between clusters, yielding a total of 17 models. The optimal model selected was the model with the best fit according to the Bayesian Information Criterion (BIC). BIC values for the models ranged from − 1503.86 to − 1571.96. We used Latent Class Analysis because it permits: (1) to identify a number of classes (subgroups) in the lifetime stressor count data that best explains the underlying scoring patterns in the data, (2) calculate the prevalence of the subgroups and (3) and estimate the individual’s probability of belonging to each subgroup^68^. In this context, Latent Class Analysis offers a more complete, nuanced and refined knowledge of participants' characteristics.

#### Analysis for lifetime stressor exposure

Descriptive statistics (means, standard errors of mean and ranges) were calculated, Table 1. Pearson parametric correlations were used to analyze associations between total lifetime stressor count, total lifetime stressor severity, mental health complaints and physical health complaints. Differences in mental health complaints and physical health complaints between stress groups were assessed using two-sample Kolmogorov–Smirnov tests. In order to examine sex differences in the total lifetime stressor count—as well as differences in the total number of lifetime stressors experienced between men and women, we ran two-sample Kolmogorov–Smirnov tests. 1-way Analysis of variance (ANOVA) were conducted to test whether total lifetime stressor count differed between groups, age or profession. Finally, two-sample Kolmogorov–Smirnov tests were carried out to investigate gender differences in the total lifetime stressor count across different primary life domains and core social-psychological characteristics.

**Table 1:** Total lifetime stressor count by participant characteristics.

Participant characteristics	N (%)	Total	Low stress		Moderate stress	
		M (SEM)	N (%)	M (SEM)	N (%)	M (SEM)
Gender						
Male	48 (23)	15.20 (1.25)	35 (23)	11.08 (0.77)	13 (25)	26.30 (2.01)
Female	157 (77)	14.82 (0.83)	119 (77)	10.04 (0.46)	38 (75)	29.78 (1.42)
Age						
18–29 years old	81 (39)	15.77 (1.11)	59 (38)	11.01 (0.63)	22 (43)	28.54 (1.93)
30–39 years old	68 (33)	14.30 (1.18)	52 (34)	9.88 (0.66)	16(31)	28.68 (1.90)
40–49 years old	34 (17)	13.82 (1.99)	26 (17)	8.57 (1.05)	8 (16)	30.87 (3.52)
50+ years old	22 (11)	15.27 (1.88)	17(11)	11.52 (1.10)	5 (10)	28.00 (3.57)
Profession						
Nursing assistant	109 (53)	14.94 (1.06)	81 (53)	9.70 (0.57)	28 (55)	30.10 (1.84)
Nurse	50 (24)	14.08 (1.28)	39 (25)	10.30 (0.82)	11 (21)	27.45 (2.12)
Physicians	41 (20)	15.39 (1.24)	31 (20)	11.77 (0.73)	10 (20)	26.60 (2.06)
Surgical technologist	5 (3)	18.60 (5.31)	3 (2)	10.00 (1.00)	2 (4)	31.50 (1.50)

(M = mean; SEM = standard error of the mean)

#### Latent class trajectory modeling

The primary purpose of our latent trajectory analysis was to identify subgroups of individuals based on their common growth trajectories over time according to their lifetime stressor count, without a priori knowledge of grouping variables. In this context, we conducted a latent trajectory analysis of the stressor data using the Flexmix package, version 2.3-13. Parameters were estimated according to the expectation–maximization algorithm and random intercepts were fit for each participant. One through ten Gaussian clusters were fit regressing lifetime stressor count data onto a seven-degree polynomial; the three-degree polynomial was determined to best fit the overall data. To provide a more accurate BIC statistic for each model, each model was estimated two times and the average BIC was taken as the BIC value for that model of interest. BIC values for the models ranged from 15,338.19 to 12,619.61. One-way ANOVA was conducted to evaluate the relationship between latent trajectories and participant’s age.

#### Analysis of the relationship between types of stress exposure and memory-guided attention

For analysis purposes, accuracy and search reaction times (RTs) for correct trials (trials performed correctly within [0.5, 4] s.) of the 24 blocks were grouped into sets of four blocks, yielding six epochs^24,59^. Two-sample Kolmogorov–Smirnov tests were used to test whether memory accuracy differed between groups. For accuracy, we carried out repeated measures ANOVA using Cue (No Cue, CC, and SR trials), Epoch (1–6), as the within-subject factors and Group (Low Stress vs. Moderate Stress) as the between-subject factor. Additionally, differences in total accuracy between groups or cue types were assessed using two-sample Kolmogorov–Smirnov tests.

To test whether RT performance on both CC and SR relative to No Cue trials differed between groups, we ran a repeated measures ANOVA using Cue (No Cue, CC, and SR trials) and Epoch (1–6) as the within-subject factors and Group (Low Stress vs. Moderate Stress) as the between-subject factor. Furthermore, we performed separate repeated measures ANOVAs for each group (i.e., Low Stress, Moderate Stress), using Cue (No Cue, CC, and SR trials during the last two epochs) as factor. ANOVAs were conducted to test differences in search RTs between CC, SR, and no-cue trials during the last two epochs. In addition, ANOVAs were run to test whether profession, gender and age were related to learning of memory cues during the last two epochs. Further, ANOVAs were conducted to test whether RT performance on CC, SR, and no-cue trials, during the last two epochs, differed between groups.

The magnitude of the cue learning was determined by the percent difference in RTs between trials with memory cues (CC or SR) and trials with No Cue, during the last two epochs. Differences in the cue learning between groups were evaluated with two-sample Kolmogorov–Smirnov tests.

To test whether SR associations were particularly elicited by the SR cue, we compared the search RTs between SR valid and SR invalid trials using a repeated measures ANOVA with Cue (SR-Valid vs. SR-Invalid) and Epoch (1–6) as the within-subject factors and Group (Low Stress vs. Moderate Stress) as the between-subject factor. Moreover, we carried out separate repeated measures ANOVAs for each group (i.e., Low Stress, Moderate Stress), using Cue (SR-Valid vs. SR-Invalid trials during the last two epochs) as factor.

For the explicit recognition test, the mean for correctly guessing target locations and target directions was computed, and the differences were evaluated with a paired t-test.

For all analyses, the alpha level was set to 0.05. Bonferroni‐correction for multiple comparisons was applied for cue (No Cue, CC, and SR trials) differences in mean RTs. Similarly, a Bonferroni test for multiple comparisons was used for latent trajectory (1–6) differences in participant’s age.

Data are reported as mean ± SEM unless stated otherwise. Throughout these analyses, sphericity violations were corrected using Greenhouse–Geisser corrections where appropriate. Alpha was set at 0.05, and partial eta-squared (*ηp2*) effect sizes were calculated with values of 0.01, 0.06, and 0.14 reflecting small, medium, and large effects, respectively. All analyses and statistical tests were performed in MATLAB (2020a, Natick, MA, USA) except where otherwise indicated.

## Results

### Latent structure of the lifetime stressor data

**Figure 2:**
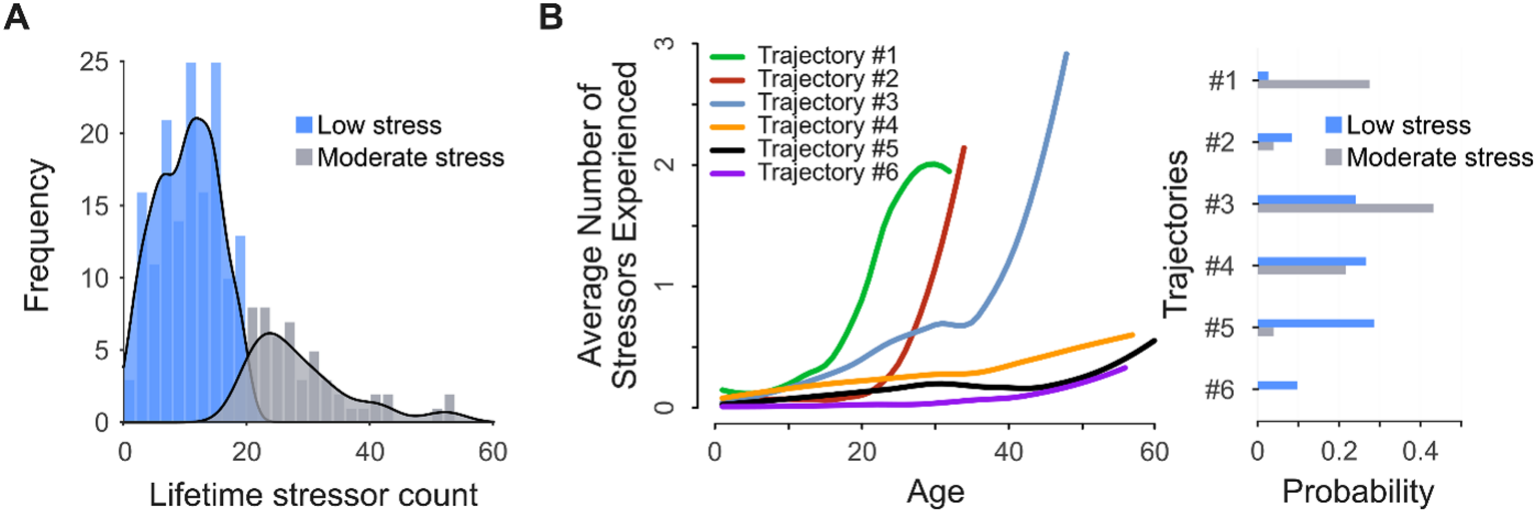
Latent structure of the lifetime stressor data. (**A**) Two latent classes best fit the underlying distribution of the overall lifetime stressor count data. On average, the low lifetime stressor exposure group (n = 154) experienced 10.27 lifetime stressors (SEM = 0.39), whereas the high lifetime stressor exposure group (n = 51) experienced 28.90 lifetime stressors (SEM = 1.18). (**B**) Left: latent trajectory analysis revealed that six latent trajectories best fit the lifetime stressor count data. Individuals in trajectory #1 (n = 18) and trajectory #2 (n = 15) showed low levels of stressor exposure through early life, but a substantial increase in stress exposure through midlife; Participants in trajectory #3 (n = 59) displayed a moderate increase in stressor exposure through early midlife followed by a substantial increase in later midlife; individuals in trajectory #4 (n = 52) showed low levels of stressor exposure through midlife, but a slight increase in stressor exposure in later life; and participants in trajectory #5 (n = 46) and trajectory #6 (n = 15) displayed very low levels of stressor exposure over the entire life course. Right: the lengths of the bars represent the probabilities of a participant with low (blue) or moderate (gray) lifetime stressor exposure falling along the corresponding trajectory.

The latent class analysis (testing the fit of 1–9 latent classes) revealed that, based on BIC, two latent classes best fit the underlying distribution of overall lifetime stressor count data—namely, a low stress group (*n* = 154; total lifetime stressor count: M = 10.27, SD = 4.95; range 0–19) and a moderate stress group (*n* = 51; total lifetime stressor count: M = 28.90, SD = 8.47; range 20–53) (see Fig. 2A). In our study, participants in the low stress group experienced an average of 10.27 lifetime stressors which correspond to a low level of lifetime stress exposure on the STRAIN (M = 13.90, SD = 6.49)^9^. Conversely, participants in the moderate stress group experienced an average of 28.90 lifetime stressors which did not correspond totally to a high level of lifetime stress exposure on the STRAIN (M = 41.25 SD = 13.25)^9^ but, it equates to a moderate level of lifetime stress exposure on the Goldfarb’s study (M = 29, range = 18–50)^41^, which is in line with previous studies of relatively low lifetime stress exposure in a similar population^69^. We thus refer to these groups as low stress group and moderate stress group, respectively.

### Lifetime stressor exposure

Participants experienced an average of 14.91 stressors over the life course (SEM = 0.70; range 0–53; possible range 0–166). The overall self-reported severity of these stressors was 33.66 (SEM = 1.64; range 0–126; possible range 0–265). As we expected, total lifetime stressor count and total lifetime stressor severity were strongly positively intercorrelated (*r* = 0.90, *p* < 0.001). In addition, lifetime stressor count was significantly associated with more self-reported current mental health complaints (*r* = 0.39, *p* < 0.001), as assessed by the Kessler 6-Item Psychological Distress Inventory (M = 10.0, SEM = 0.27; range 6–23). Similarly, greater lifetime stressor count was significantly related to greater self-reported general physical health complaints (*r* = 0.42, *p* < 0.001), as determined by the Physical Health Questionnaire (M = 30.02, SEM = 0.81; range 13–68). Importantly, the low stress group reported fewer current mental health complaints than the moderate stress group (Low-Lifetime Stressor Group: M = 9.22, SEM = 0.21, Moderate-Lifetime Stressor Group: M = 12.4, SEM = 0.62, two-sample Kolmogorov–Smirnov test: *D* = 0.33, p < 0.001). Furthermore, the low stress group reported fewer general physical health complaints in comparison to the moderate stress group (Low-Lifetime Stressor Group: M = 27.93, SEM = 0.88, Moderate-Lifetime Stressor Group: M = 36.35, SEM = 1.56, two-sample Kolmogorov–Smirnov test: *D* = 0.31, p < 0.001).

As shown in Table 1, on average, men and women did not differ in the total number of lifetime stressors experienced, (Male: M = 15.20, SEM = 1.25, Female: M = 14.82, SEM = 0.83, two-sample Kolmogorov–Smirnov test: *D* = 0.07, p = 0.67). Additionally, in the low stress group, there were no differences in the total number of lifetime stressors experienced between men and women (Male: M = 11.08, SEM = 0.77, Female: M = 10.04, SEM = 0.46, two-sample Kolmogorov–Smirnov test: *D* = 0.06, p = 0.81). Similar results were found in the moderate stress group (Male: M = 26.30, SEM = 2.01, Female: M = 29.78, SEM = 1.42, two-sample Kolmogorov–Smirnov test: *D* = 0.32, p = 0.10). Moreover, the overall lifetime stressor count was not significantly associated with participants’ age; *F*(3,201) = 0.42, *p* = 0.73, *ηp2* = 0.006. In addition, lifetime stressor count did not vary by age in the low stress group *F*(3,150) = 1.96, p = 0.12, *ηp2* = 0.037, or in the moderate stress group *F*(3,47) = 0.17, p = 0.91, *ηp2* = 0.010. Further, the total number of lifetime stressors assessed by STRAIN was not significantly associated with participants’ profession, *F*(3,201) = 0.37, *p* = 0.77, *ηp2* = 0.005. Finally, the overall stressor count was not associated with participants’ profession in the low stress group *F*(3,150) = 1.32, *p* = 0.27, *ηp2* = 0.025, or in the moderate stress group *F*(3,47) = 0.59, *p* = 0.62, *ηp2* = 0.036.

**Figure 3:**
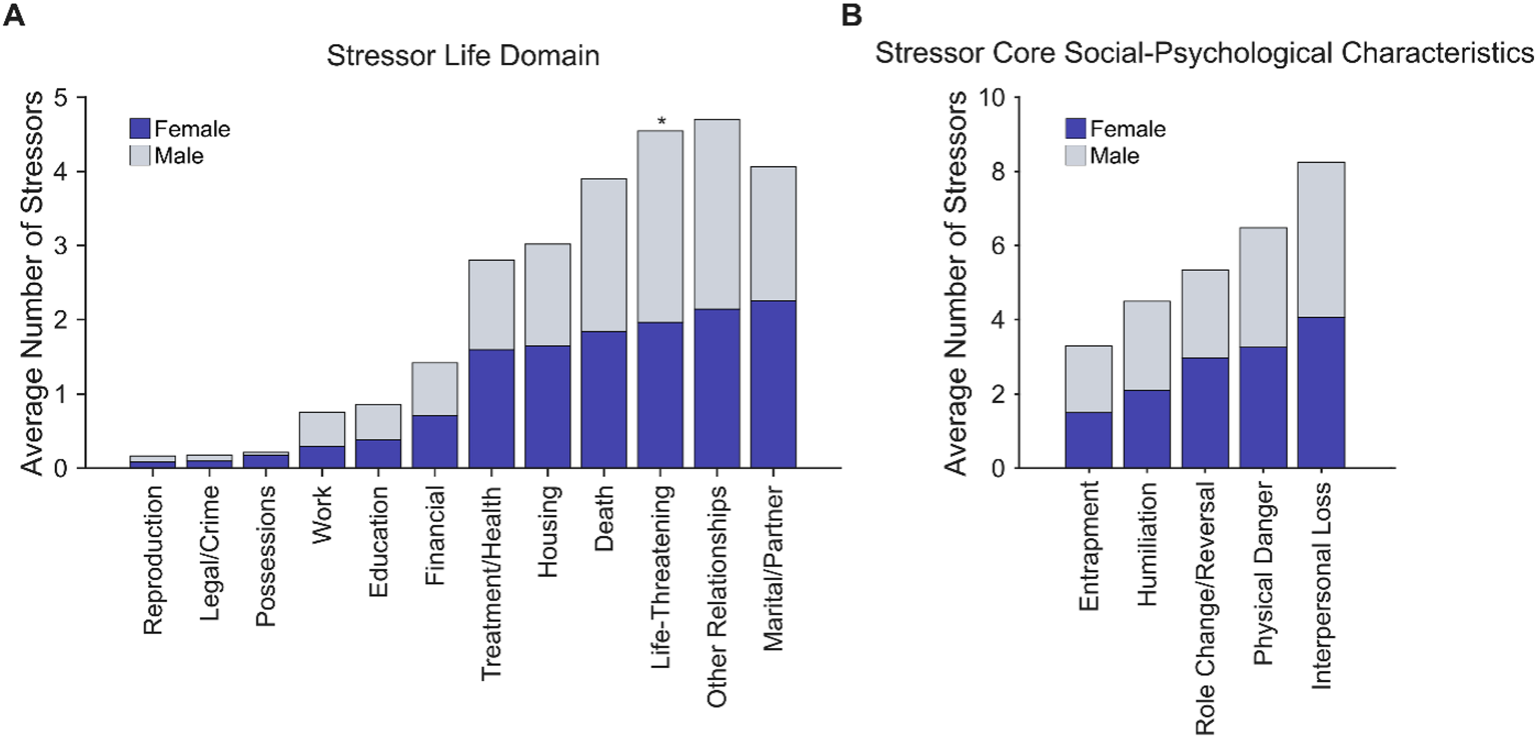
Lifetime stressor count by stressor category for males (n = 48) and females (n = 157). (**A**) Stressor Life Domains: Females (blue) reported significantly more stressors involving possessions than males (gray), while males experienced marginally more work and life-threatening situations stressors. (**B**) Stressor Core Social-Psychological Characteristics: Males and females did not differ in the number of lifetime stressors reported for each characteristic. *p < 0.05.

Taking a closer look at the stressor exposure categories revealed that, as depicted in Fig. 3A, the life stressors most commonly reported were “Other Relationships” (M = 2.23, SEM = 0.17), “Life-Threatening” (M = 2.14, SEM = 0.16) and “Marital/Partner” sources (M = 2.10, SEM = 0.16). Remarkably, males reported significantly more stressors involving life-threatening situations than females, (Male: M = 2.58, SEM = 0.35, Female: M = 1.96, SEM = 0.19, two-sample Kolmogorov–Smirnov test: *D* = 0.21, p = 0.02). Conversely, males and females did not differ in the number of lifetime stressors reported for the other characteristics (two-sample Kolmogorov–Smirnov test: ps > 0.16). With respect to the five core social-psychological characteristics, as depicted in Fig. 3B, the stressors most commonly experienced were “Interpersonal Loss” (M = 4.08, SEM = 0.18), “Physical Danger” (M = 3.24, SEM = 0.23) and “Role Change/Disruption” (M = 2.82, SEM = 0.19). Males and females did not differ in the number of lifetime stressors reported for each characteristic (two-sample Kolmogorov–Smirnov test: all ps > 0.19).

### Latent trajectory analysis of the Lifetime Stressor Data

The latent trajectory analysis revealed that the structure of the lifetime stressor data best fit six distinct trajectories over time. As shown in Fig. 2B left, trajectory 3 was the most prevalent trajectory (n = 59, 28.8%). Participants in this trajectory showed a moderate increase in stressor exposure through early midlife, followed by a substantial increase in later midlife. Individuals on trajectory 1 (n = 18, 8.8%) and trajectory 2 (n = 15, 7.3%) displayed low levels of stressor exposure through early life, but a substantial increase in stress exposure through midlife. Conversely, participants in trajectory 4 (n = 52, 25.4%) showed low levels of stressor exposure through midlife, but a slight increase in stressor exposure in later life. Similarly, individuals in trajectories 5 (n = 46, 22.4%) and 6 (n = 15, 7.3%) displayed very low levels of stressor exposure over the entire life course. Remarkably, a major proportion of participants in the Moderate Lifetime Stressor Group are part of trajectory 3 (43.1%) and trajectory 1 (27.5%). Furthermore, an important proportion of individuals in the Low Lifetime Stressor Group are part of trajectory 5 (28.6%) and trajectory 4 (26.6%), Fig. 2B Right. One-way ANOVA was conducted to evaluate the relationship between latent trajectories and participant’s age. There was a statistically significant difference between latent trajectories ANOVA (F(5,199) = 14.81, p < 0.001, ηp2 = 0.271). Post-hoc analyses revealed that the participant’s age was statistically significantly lower in the trajectory 1 (*M* = 25.33, *SEM* = 0.67, *ps* < 0.001), trajectory 2 (*M* = 29.80, *SEM* = 0.61, *ps* < 0.002) and trajectory 3 (*M* = 30.42 *SEM* = 0.81, p = 0.034) compared to the trajectory 4 (*M* = 36.46, *SEM* = 1.32), trajectory 5 (*M* = 38.84, *SEM* = 1.51) and trajectory 6 (*M* = 42.46, *SEM* = 2.05). There was no statistically significant difference in participant’s age between the trajectories 1, 2 and 3 (*ps* > 0.16), or between the trajectories 4, 5 and 6 (*ps* > 0.10), indicating that participants in the trajectories 1, 2 and 3 tended to be younger compared to participants in the trajectories 4, 5 and 6.

### Effects of lifetime stressor exposure on memory-guided attention

Consistent with previous findings^24,59^ participants were highly accurate in identifying the orientation of the target (*M* = 93.5%). In overall, accuracy did not differ between the Low and Moderate-Lifetime Stressor Group (Accuracy—Low-Lifetime Stressor Group: *M* = 92.9%, Moderate-Lifetime Stressor Group: *M* = 95.6%, two-sample Kolmogorov–Smirnov test: *D* = 0.15, p = 0.30). Further analyses reveal that there was no main effect of Lifetime Stressor Group, *F*(1, 203) = 3.28, *p* = 0.07, *ηp2* = 0.015. Similarly, there was no significant Lifetime Stressor Group × Cue interaction, *F*(2, 203) = 1.37, *p* = 0.25, *ηp2* = 0.013, Lifetime Stressor Group × Epoch interaction, *F*(5, 203) = 0.22, *p* = 0.95, *ηp2* = 0.00 or Lifetime Stressor Group × Cue x Epoch interaction, *F*(10, 203) = 0.94, *p* = 0.48, *ηp2* = 0.044, indicating that differences in RT were not due to speed-accuracy trade-offs.

For search RTs, there was no main effect of Lifetime Stressor Group, *F*(1, 203) = 0.20, *p* = 0.65, *η*_*p*_^*2*^ = 0.000, but there was a significant Lifetime Stressor Group × Cue interaction, *F*(2, 203) = 3.29, *p* = 0.03, *η*_*p*_^*2*^ = 0.031, indicating that the lifetime stressor groups differed in the use of mnemonic cues. We also found a significant main effect of Epoch, *F*(5, 203) = 7.61, *p* < 0.001, *η*_*p*_^*2*^ = 0.157, but no Lifetime Stressor Group × Epoch interaction, *F*(5, 203) = 0.23, *p* = 0.94, ηp2 = 0.005, indicating that, although the use of mnemonic cues differed for both lifetime stressor groups, the search RTs decreased during the task (see Fig. 1C). In addition, there was no significant Lifetime Stressor Group × Cue × Epoch interaction, *F*(10, 203) = 0.20, *p* = 0.99, *ηp2* = 0.009.

Importantly, the Low Lifetime Stressor Group exhibited significant differences in search RTs between CC, SR, and no-cue trials during the last two epochs, *F*(2,306) = 38.70, *p* < 0.001, *η*_*p*_^*2*^ = 0.167. Post-hoc analyses revealed that the mean RT was significantly faster on both CC (*M* = 1.002, *SEM* = 0.016, *p* < 0.001) and SR trials (*M* = 1.004, *SEM* = 0.017, *p* < 0.001) relative to No Cue trials (*M* = 1.084, *SEM* = 0.016) (see Fig. 1C), indicating that participants in the low stress group showed both significant contextual learning and stimulus–response learning. Importantly, post-hoc analyses did not show significant differences in mean RTs between SR and CC trials (*p* = 0.99). Interestingly, profession, gender, and age group were not related to learning of memory cues in the Low Lifetime Stressor Group (Cue × Profession: *F*(6,304) = 0.86, *p* = 0.51, *η*_*p*_^*2*^ = 0.016; Cue × Gender: *F*(2,300) = 0.85, *p* = 0.42, *η*_*p*_^*2*^ = 0.005; Cue × Age: *F*(6,300) = 0.47, *p* = 0.82, *η*_*p*_^*2*^ = 0.009).

Further, the Moderate Lifetime Stressor Group showed significant differences in search RTs between CC, SR and No Cue trials during the last two epochs, *F*(2,100) = 11.43, *p* < 0.001, *η*_*p*_^*2*^ = 0.186 (see Fig. 1C). Post-hoc analyses showed that the mean RTs was significantly faster for CC (*M* = 0.977, *SEM* = 0.026, *p* < 0.001) than for the No Cue trials (*M* = 1.062, *SEM* = 0.024) but no significant difference in mean RTs between SR (*M* = 1.029, *SEM* = 0.026, *p* = 0.30) and No Cue trials was found. Notably, post-hoc analyses also showed that the mean RTs was significantly faster for CC than for the SR trials (*p* = 0.04), indicating that participants in the moderate stress group showed significant contextual learning but not stimulus–response learning. Again, profession, gender, and age were not related to learning of memory cues in the Moderate Lifetime Stressor Group (Cue × Profession: *F*(6,94) = 0.29, *p* = 0.93, *η*_*p*_^*2*^ = 0.018; Cue × Gender: *F*(2,98) = 0.43, *p* = 0.64, *η*_*p*_^*2*^ = 0.008; Cue × Age: *F*(6,94) = 1.85, *p* = 0.09, *η*_*p*_^*2*^ = 0.105. Further, for search RTs during the last two epochs, we did not observe a significant main effect of Lifetime Stressor Group, F(1, 203) = 0.06, p = 0.80, *ηp2* = 0.000, or Lifetime Stressor Group × Cue interaction (1, 203) = 0.00, p = 0.90, *ηp2* = 0.000.

**Figure 4:**
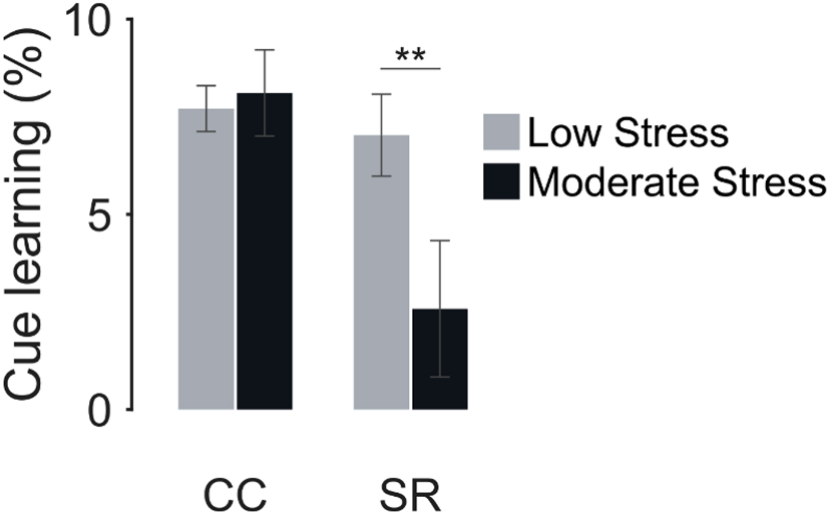
Cue learning. The percent difference between cued trials and trials with no memory cue as a function of lifetime stressor exposure group and cue type. Positive values indicate faster reaction time when using a CC or SR cue compared with No Cue. CC = context cue; SR = stimulus–response. Error bars, ± 1 SEM. *p < 0.05.

Examining the magnitude of the cue learning revealed that the lifetime stressor groups did not differ in the implicit contextual learning performance during the last two epochs (Low-Lifetime Stressor Group: *M* = 7.70, *SEM* = 0.58, Moderate-Lifetime Stressor Group: *M* = 8.10, *SEM* = 1.10, two-sample Kolmogorov–Smirnov test: *D* = 0.09, *p* = 0.82). In contrast, the SR learning performance was higher for the low lifetime stressor group than for the moderate lifetime stressor group (Low-Lifetime Stressor Group: *M* = 7.02, *SEM* = 1.04, Moderate-Lifetime Stressor Group: *M* = 2.57, *SEM* = 1.74, two-sample Kolmogorov–Smirnov test: *D* = 0.22, *p* = 0.01) (see Fig. 4). Taken together, these results suggest that moderate lifetime stressor exposure impaired the learning of SR associations but not the implicit contextual learning.

Separate analysis for SR cues revealed that the mean RT was significantly slower for SR Invalid than SR Valid trials, (SR Invalid: *M* = 1.203, *SEM* = 0.017 SR Valid: *M* = 1.084, *SEM* = 0.014, two-sample Kolmogorov–Smirnov test: *D* = 0.23, p < 0.001), indicating that SR associations were specifically evoked by the SR cue. Furthermore, we did not observe a significant main effect for Lifetime Stressor Group, *F*(1, 203) = 0.11, *p* = 0.73, *ηp2* = 0.000, or Lifetime Stressor Group × Cue [SR Valid vs. SR Invalid] interaction throughout the experiment, *F*(1,203) = 3.61, *p* = 0.058, *ηp2* = 0.017, suggesting that there were not any statistically significant performance differences between low and moderate lifetime stressor participants throughout the experiment. However, we observed a significant Group × Cue [SR Valid vs. SR Invalid] interaction during the last two epochs *F*(1,203) = 5.81, *p* = 0.01, *ηp2* = 0.027, showing that SR memory differed between the Moderate and Low-stress groups at the end of the memory test. Further analysis indicated that low lifetime stressor participants exhibited slower RTs on SR Invalid trials than on SR Valid trials during the last two epochs, *F*(1,153) = 56.21, *p* < 0.001, *ηp2* = 0.268. Conversely, moderate lifetime stressor participants did not show differences in search RTs between SR Invalid and SR Valid trials during the last two epochs, *F*(1,50) = 2.28, *p* = 0.13, *ηp2* = 0.043.

Finally, the explicit recognition test analyses revealed that neither lifetime stressor group differed from chance (25%) in recalling the ‘‘T’’ location on CC (Low Lifetime Stressor Group: *M* = 26.6%,* t*(153) = − 1.06, *p* = 0.28; Moderate Lifetime Stressor Group: *M* = 23.7%, *t*(50) = − 0.55, *p* = 0.57) or SR trials (Low Lifetime Stressor Group: *M* = 24.1%, *t*(153) = − 0.68, P = 0.28; Moderate Lifetime Stressor Group: *M* = 20.1%, *t*(50) = − 1.64, *p* = 0.10). In addition, participants in the Moderate and Low-stress groups performed at chance levels (50%) memory for the cued “T” orientation on CC (Low Lifetime Stressor Group: *M* = 47.0%, *t*(153) = − 1.87, *p* = 0.96; Moderate Lifetime Stressor Group: *M* = 45.1%, *t(*50) = − 1.77, *p* = 0.96) or SR trials (Low Lifetime Stressor Group: *M* = 23.5%,* t*(153) = − 24.24, *p* = 0.99; Moderate Lifetime Stressor Group: *M* = 24.0%, *t*(50) = − 12.40, *p* = 0.99). Taken together, these data suggest that participants in neither group could explicitly recall the learned associations. Participants in the Moderate and Low-stress groups performed at chance levels.

## Discussion

Although many studies have examined how stressor exposure relates to cognitive processes, no studies have examined the long-term effects of cumulative lifetime stressor exposure on memory in healthcare workers. To address this issue, we studied how cumulative lifetime stressor exposure related to learning for S–R and context associations under non-stressful conditions in healthcare professionals.

On average, healthcare professionals reported approximately 15 stressors over the life course, which is in line with previous studies of relatively low overall lifetime stress exposure in similar populations^69^, other relationships, life-threatening and marital/partner resources were the three life stressors most commonly reported by participants, which emphasizes the importance of interpersonal relationships as a source of stress^70^. Similarly, Interpersonal Loss, Physical Danger and Role Change/Disruption were the three social-psychological characteristics most commonly reported. As expected, experiencing more total lifetime stressors was both related to experiencing more mental health and physical health complaints, which agrees with recent evidence indicating that cumulative lifetime stressor exposure is associated with physical health problems^7–9,11,12^ and mental disorders^71,72^. Importantly, in our sample, the overall lifetime stressor count was not significantly associated with participants’ gender, age, or profession.

Among our sample, an estimated 16.1% of such healthcare professionals had sustained trajectories of low levels of stressor exposure through early life, but a substantial increase in stress exposure through early midlife. Additionally, an estimated 28.8% of healthcare professionals had sustained trajectories of moderate increase in stressor exposure through early midlife followed by a substantial increase in later midlife. On the contrary, 55.1% of healthcare professionals had sustained trajectories of low levels of stressor exposure over the entire life course. Our results are in contrast with a recent study in which 82.4% of individuals belonging to the general population exhibited low levels of stress exposure over the entire life course, while just 17.6% of individuals exhibited a substantial increase in stress exposure over time^9^). Taken together, the above suggests that a greater proportion of healthcare professionals exhibited low levels of stressor exposure through early life, but a substantial increase in stress exposure through midlife in comparison with the general population. Notably, a major proportion of healthcare professionals in the Moderate Lifetime Stressor Group (43.1%) had sustained trajectories of moderate increase in stressor exposure through early midlife, followed by a substantial increase in later midlife.

As expected, we found that healthcare professionals with moderate cumulative lifetime stressor exposure exhibited impaired learning for S–R associations relative to those with low cumulative lifetime stressor exposure. In contrast, learning for context associations was not affected in healthcare professionals with low or moderate cumulative lifetime stressor exposure. These results are thus the first to show that moderate, but not low, cumulative lifetime stressor exposure significantly reduces the engagement of striatum dependent S–R learning strategies under non-stressful conditions.

Interestingly, we observed that low cumulative lifetime stressor exposure did not impair the learning for context and S–R associations. This finding is consistent with recent reports indicating that, under non-stressful conditions, healthy individuals can use context hippocampus-based and S–R striatum-based learning strategies^24,59^. Moreover, the present findings corroborate previous studies showing that, under certain circumstances, hippocampal and striatal memory systems can concurrently acquire information^73^ and operate independently^34,35^. However, our current finding might appear to be in conflict with recent data showing that low levels of lifetime stress impair striatum-dependent S–R learning^41^. These discrepancies are most likely due to the differences that exist between the ages of participants. The average age in their study^41^ was 19.70 years, 14 years younger than the average age in our study sample (34.23 years). Perhaps, the decreasing self-perceived stress with age^74^, age-related stress-coping mechanisms^75^, or endogenous factors may also play a role in the learning of striatum-dependent S–R. Future studies that include participants from a broad age range would be valuable. Additionally, the missing low cumulative lifetime stressor exposure effect on striatum-dependent S–R learning might be also explained by the differences in the number of stressors and stressor severity experienced in the low stress group. Compared with the present study, Goldfarb and colleagues^41^ was based on a sample with a smaller average of stressors experienced (3.66 vs. 10.23 stressors) and lower overall severity (6.46 vs. 24.09). Possibly, low and very low cumulative stress exposure could differentially modulate the striatum-dependent S–R learning. In this context, it is possible that further segmenting cumulative life stress levels into more narrow intervals would reveal interesting differential relationships between levels of cumulative lifetime stressor exposure and striatum-dependent S–R learning.

The impairing effect of moderate cumulative lifetime stress on S–R learning, but not on context learning, is in line with prior research showing the engagement of hippocampal over striatal learning strategies under both non-stressful conditions and lower cortisol levels^26,27^. Since high glucocorticoid levels facilitate S–R learning^31^ and low glucocorticoid levels promotes hippocampus-dependent learning^25,35^, the negative effects of moderate cumulative lifetime stress on S–R striatum-dependent learning observed here may be due in part to the fact greater cumulative lifetime stressor exposure can lead to reduced cortisol response^6,10,18^ and decreased cortisol availability^19^, which, in turn, would interfere with S–R learning performance. Accordingly, there is evidence that high cumulative stress (e.g., increasing levels of chronic stress over time) is associated with flatter diurnal cortisol slopes, reduction in morning cortisol levels and an increase in evening cortisol levels^76^. Since our participants principally performed the learning task in the morning, then we would expect that participants with moderate levels of stress would show lower cortisol levels than those with low stress. It is, however, important to note that there are several factors that could influence diurnal cortisol levels in healthcare professionals, including misalignment of their cortisol circadian rhythm due to nonstandard work schedules^77,78^. Finally, because in our study did not include measurements of baseline cortisol levels, it cannot be concluded that decreased cortisol availability contributes to performance deficits in individuals with moderate cumulative lifetime stress. Future studies are needed to draw firm conclusions.

Our finding that moderate cumulative lifetime stress impairs S–R learning performance is in disagreement to previous reports in which chronic stress exposure facilitates the engagement of striatum over hippocampus-dependent memory system but were not impaired in learning^44^. One reason may be the time-window for examining the stressor exposition. In the current study we assessed participants’ cumulative exposure to stressors that they could have experienced over the entire life course, whereas in the study that found a facilitating effect of high levels of stress exposure on S–R learning, only the stressors experienced by the participants within the 3 months prior to testing were assessed. Recent life stress exposure may transiently facilitate the expression of striatum-dependent strategies, at the expense of hippocampus-dependent strategies, without affecting striatum or hippocampus-dependent learning (current chronic stress levels)^44^, while moderate cumulative lifetime stress may exert a perdurable negative effect on striatum-dependent learning (stress history). Importantly, cumulative stress exposure tends to be a better indicator of cumulative effect on biological processes that underlie disease than chronic stress measured alone^9,10^.

Previous studies showed that pre-learning stress impairs hippocampus-dependent learning^28–30^. Here, we observed that the overall magnitude of contextual learning in individuals with moderate cumulative lifetime stress exposure was not significatively different from those who had experienced lower levels of stress. This apparent discrepancy can be explained by the timing of the stressor. In the studies that found an impairing effect of stress on hippocampus-dependent memory, participants began the memory test minutes after the stress manipulation, whereas in the current study, participants began the memory test without stress manipulation, but with their long-term history of stress exposure. Since the effects of stress on hippocampus-dependent memory rely on the temporal proximity of the stressor to the learning experience^28,29^, we do not expect significant effects of cumulative lifetime stress exposure on the overall magnitude of contextual learning under non-stressful conditions. Additionally, it has been observed that the most pronounced impact of stress on hippocampus is during the early life period^79^. Because a major proportion of individual in the Moderate Lifetime Stressor Group had sustained trajectories of moderate increase in stressor exposure through early midlife, we do not envisage impairment of hippocampus-dependent learning.

Since previous studies suggest that cumulative lifetime stressor exposure is related to decreased cortisol response^6,10,18^ and reduced basal cortisol secretion^19^ and consequently, acquisition of context and S–R associations could be affected initially biasing the memory expression, we were interested in evaluating the effects of cumulative lifetime stressor exposure on learning rather than on memory expression itself. Accordingly, we used a dual solution task to measure learning of these associations separately, without using a probe test, in which only one memory system drives performance^80^. The main advantage of this approach consists in the possibility to assess learning for context and S–R associations in the same task and independently of memory expression. However, if the task involves concurrent (close temporal proximity) acquisition of context hippocampus-based and S–R striatum-based learning, then these systems may couple functionally, and the learning will reflect this coupling^81^. Importantly, despite that we did not evaluate context and S–R memory, it has been suggested that differences in stimulus–response learning are related to differences in stimulus–response memory in the dual-solution task^41,80^.

Finally, some potential limitations of our studies should be acknowledged. First, although we assessed cumulative lifetime stressors using a well-validated self-report instrument that has been shown to be insensitive to personal processes and social desirability, we cannot rule out the possible influence of self-reporting biases in these effects. For example, more recent stressors could still have influenced participants' reports of the stressors they experienced across the lifespan. Future studies should thus prevent the influence of self-reporting biases using stress assessment methods that ensure an independent estimation of stressor count. Second, we recruited healthcare workers for this sample, and additional research is needed to investigate the generalizability of the current results to other populations. Finally, although our results suggest a putative link between cumulative lifetime stress and reduced striatum dependent S–R learning, we did not assess S–R learning-related changes in cortisol and brain activity. Future research is thus needed to examine how the dissociations reported here are both driven by competitive or cooperative interactions between the striatum and hippocampus, and modulated by neuroendocrine activity.

Despite these limitations, the present data show that individuals exposed to a moderate number of major life stressors over the lifespan exhibit impaired learning for S–R, but not for contextual, associations among healthcare professionals. These results thus provide novel evidence that the cumulative lifetime stressor exposure exerts a negative effect on human striatum dependent S–R learning under non-stressful environmental conditions. Understanding the underlying neuroendocrine mechanisms of this effect may have important implications for human cognition and disease.

## Data Availability

The datasets used and/or analyzed during the current study available from the corresponding author on reasonable request.
